# Research progress on the application of anti-gravity treadmill in the rehabilitation of Parkinson’s disease patients: a mini review

**DOI:** 10.3389/fneur.2024.1401256

**Published:** 2024-05-31

**Authors:** Yalin Zheng, Yu Shen, Renzhi Feng, Weiyin Hu, Peng Huang

**Affiliations:** School of Sports Medicine and Rehabilitation, Beijing Sport University, Beijing, China

**Keywords:** Parkinson’s disease, anti-gravity treadmill, rehabilitation, postural gait abnormalities, balance and physical activity, cognitive impairment

## Abstract

Parkinson’s disease (PD) is a progressive neurodegenerative disorder characterized by motor and non-motor symptoms. It is the second most common chronic progressive neurodegenerative disease. PD still lacks a known cure or prophylactic medication. Current treatments primarily address symptoms without halting the progression of PD, and the side effects of dopaminergic therapy become more apparent over time. In contrast, physical therapy, with its lower risk of side effects and potential cardiovascular benefits, may provide greater benefits to patients. The Anti-Gravity Treadmill is an emerging rehabilitation therapy device with high safety, which minimizes patients’ fear and allows them to focus more on a normal, correct gait, and has a promising clinical application. Based on this premise, this study aims to summarize and analyze the relevant studies on the application of the anti-gravity treadmill in PD patients, providing a reference for PD rehabilitation practice and establishing a theoretical basis for future research in this area.

## 1 Introduction

Parkinson’s disease (PD) is a progressive neurodegenerative disorder characterized by motor and non-motor symptoms (1). It ranks as the second most common chronic progressive neurodegenerative disease (2). With a 2.5-fold increase in prevalence over the past 30 years, the current overall global prevalence exceeds 6 million (3). Tremors at rest, rigidity, bradykinesia, postural instability, and gait disturbances constitute the primary motor signs of PD, leading to progressive impairment. Freezing of gait (FOG) is considered one of the most disabling gait disorders in patients with Parkinson’s disease and is defined as “a brief, episodic absence or marked reduction of forward progression of the feet despite the intention to walk” (4). Patients often describe it as if they cannot move their feet while their upper body continues to maintain its original motion (5). A systematic review has shown that treadmill training enhances the fitness of PD patients and represents the exercise therapy intervention with the most significant impact on UPDRS scores (6). Research has revealed that 47% of PD patients exhibit FOG (1), which is a major contributing factor to patients’ mobility issues and elevated fall risk (7). Furthermore, as the disease progresses, the non-motor symptoms of PD deteriorate. These symptoms are primarily associated with a diminished sense of smell, constipation, urinary dysfunction, cognitive impairment, upright hypotension, memory loss, depression, and sleep disorders (8, 9), leading to a lower quality of life for patients.

Clinically, treatments include Deep Brain Stimulation (DBS) and dopamine-based pharmacologic therapies (10). Although DBS is a highly effective treatment for PD (11, 12), its invasiveness, risk of postoperative complications, and expensive cost make it less desirable than other options. Pharmacologic therapies are the main treatment for PD, including levodopa, methyldopa, and silymarin, which can ameliorate patients’ motor symptoms. However, the efficacy of dopaminergic medicines for PD will eventually wear off and reveal side effects. Additionally, existing dopaminergic therapies can only alleviate symptoms and do not stop the progression of the disease, and may lead to serious complications such as on–off phenomenon and L-DOPA-induced dyskinesia, further complicating the treatment process (13). Despite the application of pharmacologic treatments, gait impairment in PD patients remains (14). Studies have demonstrated a higher risk of developing FOG in patients prescribed levodopa compared to those who were not (15).

Conversely, physical therapy offers a lower risk of side effects and potential cardiovascular benefits, potentially providing greater benefits to patients (16). Aquatic exercise therapy, which has received much attention in recent years, is a training method that utilizes the properties of water, such as buoyancy, turbulence, hydrostatic pressure, and resistance to enable patients to perform exercises in water, thereby improving muscle strength, endurance, and coordination (17). A systematic review revealed that aquatic exercise therapy significantly enhanced patients’ static balance compared to land-based exercise (18). While aquatic exercise therapy has beneficial effects on gait, balance, and mobility in the early stages of PD that are equivalent to those of land-based physical therapy, the ideal exercise dose, training content, and effective duration are still up for debate (19). A recent study found that 36.5% of individuals with PD experienced dyspnea in water and that they are potentially at risk of drowning due to their asymmetrical motor characteristics, which make it difficult to execute complex movements (20). Furthermore, approximately 30% of patients will develop dementia and severe executive dysfunction, which impairs semantics, situational memory, and visuospatial perception and construction (3). This will impose stricter requirements on the venue, hydrotherapy equipment, and supervisory staff. Therefore, there are limitations in the application of aquatic exercise therapy for elderly patients with mid to late-stage PD. However, the anti-gravity treadmill is more comfortable than traditional support belt weight-loss training systems, allowing patients to focus more on correct gait, and can provide more comfortable aerobic exercise and walking (21). Based on this premise, this review summarizes and analyzes the relevant studies on the application and mechanism of the anti-gravity treadmill in PD patients aiming to provide a reference for PD rehabilitation practice and establish a theoretical basis for future research in this area.

## 2 Anti-gravity treadmill

The use of treadmills in gait analysis and rehabilitation training approaches has been widespread, as they facilitate the execution of various gait cycles while maintaining the patient’s natural gait performance (22). One of the primary therapeutic objectives of PD is to maintain mobility and physical function. The treadmill makes it possible to train continuously and rhythmically, which creates the perfect environment for mimicking the precise motions required for a stabilized gait. However, there are safety concerns while using typical treadmills since individuals with mid to late-stage Parkinson’s disease (PD) often experience cognitive impairment, impaired somatic function, and a higher risk of falling (23).

Extended exposure to microgravity during spaceflight can lead to serious deterioration of astronauts’ physical functions (24). The anti-gravity treadmill, developed by the National Aeronautics and Space Administration (NASA), is a novel body weight-supported walking training system that utilizes air pressure differences in space to mimic the Earth’s gravitational pull to prevent astronauts from losing bone mass and muscle deterioration. It can effectively minimize the effects of gravity on their bodies. Today, it is increasingly utilized in sports and clinical settings, including patients with running injuries (25), osteoarthritis (26), cerebral palsy (27, 28), pelvic stress fractures (29), etc. The anti-gravity treadmill is simple to operate and requires relatively little from the patient. The patient wears specially designed shorts connected to an airbag that aligns with the waist. The system calibrates the airbag to the patient’s size and weight. When air is blown in, the air pressure of the pneumatic system reduces the force of gravity in 1% increments, up to 80%, reducing the risk of falling to almost zero and providing a high degree of safety. It not only alleviates the patient’s fear but also helps to improve patient motivation and compliance (26, 30). The patient’s lower limbs can swing more naturally while maintaining gait mechanics because of the device’s decentralized and uniform weight support, which is beneficial for neuromuscular re-education and more by physiological movement patterns (31). The anti-gravity treadmill not only produces similar effects as aquatic exercise therapy but also can accurately and gradually adjust the patient’s weight bearing. The U.S. Food and Drug Administration has approved the use of the anti-gravity treadmill in outpatient medical and physical therapy clinics, and it is safe for intracranial and systemic hemodynamic parameters (32).

## 3 The application of anti-gravity treadmill in the rehabilitation of Parkinson’s disease patients

### 3.1 Improving postural gait abnormalities in Parkinson’s disease patients

In the early-stage PD, patients’ postural abnormalities are mainly characterized by forward or lateral trunk flexion during standing, and head and neck flexion (33, 34), when functional deficits are relatively minor significant milestone indicating the progression of the disease is the onset of freezing gait. A study of the kinetics of the turning process in 23 PD patients revealed that individuals with PD turn more slowly and require more steps to complete the turning maneuver (35). Mortality tends to rise when patients develop postural and gait symptoms——mainly due to falls, hip fractures, etc. Typically, an individual with PD experiences a seven-year decrease in life expectancy after they start to fall (36). The pathophysiologic causes and determinants of freezing gait are multifaceted. Fasano et al. (37) found that the site of the lesion leading to freezing gait is functionally connected to a focal area in the dorsomedial cerebellum; Lewis et al. (38) proposed that there are different pathophysiologic circuits in the brain and that alterations or damage to individual circuits can imbalance the entire neural network, ultimately leading to the development of a freezing gait.

PD patients may exhibit excessive muscle activity in the muscles governing their knee joint (39). Patients can improve their gait with most forms of physical activity (6). Exercise in a body weight-supported environment promotes normalized activation of the extensor muscles of the lower extremities in PD patients, and extensor off-activation time and peak muscle activation will normalize when weight support approaches 80% (39). PD patients may see a reduction in over-activation of their extensor muscles in a low-gravity environment which can help patients with their postural irregularities and efficiently manage the tone of their lower limb muscles. PD patients benefit from treadmill training which improves their gait and walking ability, including stride length and walking speed (40, 41). A result of a randomized controlled study showed that 8 weeks of treadmill training significantly improved gait and postural stability in PD patients. Rose et al. (42) reported that PD patients had a 10.6% increase in mean walking distance after 8 weeks of aerobic exercise on anti-gravity treadmill. Baizabal-Carvallo et al. (43) investigated the effects of 4 weeks of anti-gravity treadmill training in PD patients with moderate-to-severe gait freezing, revealing increased gait speed and stride length. 84% of the patients reported moderate or significant improvement in gait, indicating potential benefits of the anti-gravity treadmill even in PD patients with low MoCA scores (i.e., cognitive deficits are present). The pathophysiology of freezing gait is intricate, and the neurophysiologic mechanisms by which anti-gravity treadmills improve freezing gait are not yet fully comprehended. However, some researchers have hypothesized that under low-gravity conditions, decreased ground reaction forces and reduced proprioceptive inputs may lead to better integration of neuronal motor circuits with certain motor abilities in PD patients and that patients may also be able to enhance neuronal compensatory mechanisms by improving their gait (43), which may lead to an improvement in freezing gait. PD patients who are older may find it difficult to run or increase their walking speed without fear of falling when using the traditional treadmill. Steadily raising their weight on the anti-gravity treadmill until they can support their entire body weight, enables them to practice exercises and gradually boost their self-confidence in their athletic ability.

### 3.2 Improving balance and physical activity in Parkinson’s disease patients

Physical activity is defined as any bodily movement caused by skeletal muscles that results in energy expenditure (44). According to studies, those who engage in regular, prolonged, moderate-to-intense physical activity have a 40% lower chance of acquiring PD than people who lead sedentary lives (45). Muscle weakness and other motor deficits significantly hinder the ability of people with PD to participate in physical activities, resulting in significantly lower levels of physical activity (46). PD patients who fall frequently also have trouble performing daily tasks and have a strong dread of falling again (47). If balance deficits and fear of falling persist or worsen, this may prevent PD patients from performing activities of daily living. Matinolli et al. (48) indicated that increased disease severity and postural sway seem to be the most important independent risk factors for the occurrence of falls in PD patients. A recent survey of falls in PD patients showed that falls were twice as frequent as in age-matched older adults (49).

In PD-affected mice, exercise has been demonstrated to boost dopamine receptor DA-D2R (50), and comparable outcomes have been seen in PD patients (51). Research has shown that after 4 weeks of anti-gravity training, the Test of Timed Up and Go (TUG) was shortened by 7 s in PD patients (43), which significantly improved the dynamic balance and reduced the risk of falls. In the sagittal and cross-sectional planes, PD patients demonstrated increased performance in executing dynamic balance-related tasks after 8 weeks of anti-gravity treadmill training, as evidenced by a 24% improvement in the completion time of the five sit-to-stand tests (52). However, this study had a small sample size and only male PD patients. Further studies are required to investigate the effects of an anti-gravity treadmill on dynamic balance and physical activity in PD patients in a larger sample size.

### 3.3 Improving cognitive impairment in Parkinson’s disease patients

One of the most common non-motor symptoms of PD is cognitive dysfunction; at the time of diagnosis, 20%–33% of individuals had modest cognitive impairment (53). Research has demonstrated a significant correlation between motor and motor learning deficits and cognitive dysfunction in PD patients (54, 55). Nevertheless, bradykinesia is a risk factor for mild cognitive impairment in PD patients and this population is at higher risk for cognitive impairment (56). Patients with early PD had significantly lower serum BDNF levels compared to healthy controls (57). However, experimental animal studies have shown that the treadmill exercise increased the levels of BDNF and GDNF in the striatum of a rat model of PD (58, 59), upregulated the levels of Nrf2 and γGCLC, and reversed dopaminergic neurodegeneration of the substantial nigrastrata (60). They exert neurorestorative and neuroprotective effects by modulating neurotrophic factors to stimulate synapse formation and angiogenesis, inhibit oxidative stress, and improve mitochondrial function. Johansson et al. (61) demonstrated that aerobic exercise reduces brain atrophy, stabilizes motor processes, and enhances cognitive performance in PD patients by stimulating functional and structural plasticity in cortical striatal sensorimotor and cognitive control networks, while Fisher et al. (51) showed that exercise increases the neuroplasticity of dopaminergic signaling. A review noted that in PD patients, strength, flexibility, motor control, and cognitive abilities are consistently improved by exercise, resulting in better functional abilities (62). These findings are consistent with those of PD animal models.

Besides providing similar benefits to aerobic training on a conventional treadmill, the anti-gravity treadmill is also more applicable to elderly, less mobile, and mid-to-late-stage PD patients. However, there are still gaps in research on exercise therapy to improve cognitive deficits in patients with PD, and more large-sample, multicenter, randomized controlled studies need to be included in the future to guide a more diverse range of rehabilitative treatments for PD patients.

### 3.4 Improving the quality of life and sleep disorders for Parkinson’s disease patients

The motor and non-motor symptoms of PD significantly impact the quality of life, including physical, mental, emotional, social, and economic aspects. A meta-analysis revealed that PD patients scored poorer on most measures of quality of life than healthy controls, particularly in terms of their physical and mental well-being (54). Numerous studies have shown that depression is the most significant factor affecting quality of life (63, 64), accounting for 60% of the quality of life impairment (65). Anxiety, apathy, and pain are also associated with quality of life in PD patients (66) and have a greater impact than motor symptoms. Patient’s reduced quality of life not only hurts their treatment plan but also shortens their life expectancy (67).

Anti-gravity treadmill training can improve the quality of life and negative emotions in PD patients. Research reveals that after 8 weeks of anti-gravity treadmill exercise, PD patients experienced a significant improvement in their PDQ-39 index, with an average increase of 8.3 points in the composite index, representing a 32.6% improvement compared to healthy controls (42). Atan et al. (68) found significant improvements in energy and physical activity subgroup scores on the Nottingham Health Profile (NHP) after a 6-week weight support training program for PD patients. Animal experiments demonstrated that treadmill exercise prevented depressive-like behavior and restored the levels of proBDNF, BDNF, and TrkB in the striatum and hippocampus of mice with PD, suggesting the effectiveness of exercise training in neuroprotection of the striatum and hippocampus (69). However, there is considerable variation in the scales currently used in different studies investigating quality of life, depression, and anxiety in PD patients. For example, several research measures like the Hamilton Depression Rating Scale (HDRS) and the Beck Depression Inventory (BDI) used for assessing depression in PD patients have not received approval for use within this patient population (70). To assure high-quality study data and enable comparability among related studies, we strongly advise researchers to use officially recommended or suggested scales in their future research, as well as provide details about the statistical procedures used.

Given the distress caused by motor and non-motor symptoms in PD patients, along with the prevalence of anxiety, depression, and adverse reactions to medications, they are susceptible to sleep disorders, which can negatively affect the regression of the disease. Sleep represents the most common non-motor symptom of PD, with a prevalence of approximately 47.66%–89.10% (71). Research indicates that both physical activity levels and ideal sleep patterns are independently associated with a lower risk of PD, whereas individuals with high physical activity levels and ideal sleep patterns have the lowest risk of developing PD. Therefore, enhancing physical activity levels and sleep quality may be promising targets for intervention to prevent PD (72). A study noted that treadmill training increased cortical striatal neuroplasticity and dopamine release, thereby improving sleep quality (73). Treadmill training represents the most straightforward type of aerobic exercise, and the anti-gravity treadmill, amalgamating the benefits of the traditional treadmill and is more practical and safer in PD, can be effective in improving physical activity levels and sleep disorders in PD patients, thereby improving their quality of life. However, other studies with larger sample sizes and higher quality are needed to validate the effects of the anti-gravity treadmill on the quality of life of PD patients.

## 4 Conclusion and future directions

Recent studies have demonstrated that anti-gravity treadmill training effectively ameliorates postural gait abnormalities in PD patients, mitigates fall risks, and enhances both balance function and mobility. Moreover, anti-gravity treadmill training yields substantial benefits for PD patients, addressing cognitive deficits, sleep disorders, and overall quality of life. This study provides a reference for the rehabilitation practice of PD patients and provides new rehabilitation ideas for better returning to family and society.

However, it is regrettable that there remains a paucity of studies investigating the application of anti-gravity treadmills in the rehabilitation of PD and other neurodegenerative diseases. Existing research has primarily emphasized the enhancement of gait abnormalities and balance function in Parkinson’s disease patients, overlooking critical issues like cognitive deficits and quality of life aspects, which significantly impact Parkinson’s disease patients. Consequently, further research efforts should encompass large-scale, multicenter, randomized controlled trials to address these gaps and offer comprehensive guidance on PD patient rehabilitation.

In addition, exercise prescription for anti-gravity treadmill is also a direction for future research. It includes exercise duration, exercise intensity, exercise frequency, percentage of weight loss, and dual- or multitasking needs to be further investigated. Furthermore, there is inadequate evidence-based medical support for determining the optimal training regimen for PD patients with varying disease processes, necessitating further exploration into the effectiveness disparity among different exercise regimens and their duration of efficacy. Future trials should adopt a rigorous study design, use both subjective and objective outcome measures, and conduct long-term follow-ups to determine more effective strategies for anti-gravity treadmill management in PD.

In conclusion, the anti-gravity treadmill stands as a burgeoning rehabilitation device that gradually reduces gravity, leading to weight loss akin to aquatic therapy but with enhanced safety. Its potential to enhance daily activity in PD patients and its favorable clinical application outlook make it a safe and efficient exercise alternative, particularly beneficial for elderly individuals with advanced PD and restricted mobility necessitating comprehensive supervision.

## Author contributions

YZ: Project administration, Writing – original draft, Writing – review & editing. YS: Writing – original draft. RF: Writing – review & editing, Writing – original draft. WH: Writing – review & editing, Writing – original draft. PH: Supervision, Writing – review & editing.
